# Different dose regimes and administration methods of tranexamic acid in cardiac surgery: a meta-analysis of randomized trials

**DOI:** 10.1186/s12871-019-0772-0

**Published:** 2019-07-15

**Authors:** Jingfei Guo, Xurong Gao, Yan Ma, Huran Lv, Wenjun Hu, Shijie Zhang, Hongwen Ji, Guyan Wang, Jia Shi

**Affiliations:** 1 Department of Anesthesiology, Fuwai Hospital, National Center for Cardiovascular Diseases, Chinese Academy of Medical Sciences, and Peking Union Medical College, No.167 Beilishi Road, Xicheng district, Beijing, China; 2 Department of Blood Transfusion, Fuwai Hospital, National Center for Cardiovascular Diseases, Chinese Academy of Medical Sciences, and Peking Union Medical College, No.167 Beilishi Road, Xicheng district, Beijing, China; 3 Operating room, Fuwai Hospital, National Center for Cardiovascular Diseases, Chinese Academy of Medical Sciences, and Peking Union Medical College, No.167 Beilishi Road, Xicheng district, Beijing, China; 4 Department of Anesthesiology, The 305th Hospital of the Chinese People’s Liberation Army, No.13 Wenjin Road, Xicheng district, Beijing, China; 5 Department of Anesthesiology, Wu’an First People’s Hospital, Kuangjian Road, Handan, Hebei Province China

**Keywords:** Tranexamic acid, Cardiac surgery, Seizure, Dose regimen

## Abstract

**Background:**

The efficacy of tranexamic acid (TXA) to reduce perioperative blood loss and allogeneic blood transfusion in cardiac surgeries has been proved in previous studies, but its adverse effects especially seizure has always been a problem of concern. This meta-analysis aims to provide information on the optimal dosage and delivery method which is effective with the least adverse outcomes.

**Methods:**

We searched Cochrane Central Register of Controlled Trials, MEDLINE and EMBASE for all relevant articles published before 2018/12/31. Inclusion criteria were adult patients undergoing elective heart surgeries, and only randomized control trials comparing TXA with placebo were considered. Two authors independently assessed trial quality and extracted relevant data.

**Results:**

We included 49 studies with 10,591 patients into analysis. TXA significantly reduced transfusion rate (RR 0.71, 95% CI 0.65 to 0.78, P<0.00001). The overall transfusion rate was 35%(1573/4477) for patients using TXA and 49%(2190/4408) for patients in the control group. Peri-operative blood loss (MD − 246.98 ml, 95% CI − 287.89 to − 206.06 ml, P<0.00001) and re-operation rate (RR 0.62, 95% CI 0.49 to 0.79, P<0.0001) were also reduced significantly. TXA usage did not increase risk of mortality, myocardial infarction, stroke, pulmonary embolism and renal dysfunction, but was associated with a significantly increase in seizure attack (RR 3.21, 95% CI 1.04 to 9.90, *P* = 0.04).The overall rate of seizure attack was 0.62%(21/3378) for patients using TXA and 0.15%(5/3406) for patients in the control group.

In subgroup analysis, TXA was effective for both on-pump and off-pump surgeries. Topical application didn’t reduce the need for transfusion requirement, while intravenous delivery no matter as bolus injection alone or bolus plus continuous infusion were effective. Intravenous high-dose TXA didn’t further decrease transfusion rate compared with low-dose regimen, and increased the risk of seizure by 4.83 times. No patients in the low-dose group had seizure attack.

**Conclusions:**

TXA was effective in reducing transfusion requirement in all kinds of cardiac surgeries. Low-dose intravenous infusion was the most preferable delivery method which was as effective as high-dose regimen in reducing transfusion rate without increasing the risk of seizure.

**Electronic supplementary material:**

The online version of this article (10.1186/s12871-019-0772-0) contains supplementary material, which is available to authorized users.

## Background

Tranexamic acid(TXA)is a lysine analogues which acts principally by blocking the lysine binding sites on plasminogen molecules, inhibiting the formation of plasmin and therefore inhibiting fibrinolysis [1]. The efficacy of TXA to reduce perioperative blood loss and allogeneic blood transfusion has been studied extensively. The latest Cochrane review on TXA was published in 2011 [2], the author concluded that TXA was effective in reducing blood loss and transfusion requirement in multiple kinds of surgeries including cardiac surgery, and appeared to be free of serious adverse effects. However, as an anti-fibrinolytic agent, the prothrombotic effect has always been a concern. TXA could potentially increase the risk of myocardial infarction, stroke, and other thrombotic complications, and has later been shown to increase the risk of neurologic events [3, 4], especially seizures [5].

In 2017, Myles et al. published a study on TXA usage in cardiac surgery [6]. In this randomized controlled trial including 4631 patients, the author found that seizures occurred in 0.7 and 0.1% in TXA and placebo group respectively (*P* = 0.002). This recent study draws further attention to the dosage regimen of TXA, since adverse effects including seizure are possibly dosage-related. The study used a high-dose regimen, in which either 50 mg/kg or 100 mg/kg of TXA was delivered for each patient. There is a possibility that lower dose of TXA can be equally effective while causing less adverse effects. In fact, TXA plasma concentrations required to suppress fibrinolysis and plasmin-induced platelet activation are merely 10 and 16 μg/ml, respectively [7, 8]. This relatively low plasma concentration can be reached in cardiac surgery when 10 mg/kg of TXA is administered as a bolus then followed by continuous infusion of 1 mg kg/h and 1 mg/kg in CPB [9]. But another potential mechanism of TXA action might be the increase in thrombin formation, which requires concentrations more than 126 μg/ml to be effective [10, 11]. 30 mg/kg of TXA administered as a bolus followed by 16 mg/kg/h and 2 mg/kg in CPB prime solution was able to maintain the plasma concentration above 114 μg/ml [9].

The dosage and delivery methods of TXA in cardiac surgery has long been a problem of debate. Drug administration varied significantly across studies, and no agreement has been reached concerning the following issues: what is the ideal dosage of TXA, whether TXA should be delivered intravenously or topically, whether continuous infusion or bolus injection should be used. This study aims to summarize evidence from all trials published before 2019 and try to provide information on the optimal dosage and delivery method which is effective with the least adverse outcomes.

## Methods

### Criteria for considering studies for this review-the PICO framework

#### Patient

Adults who underwent elective heart surgery were included in the study. Trials on urgent cases or on children were excluded. We included both on and off pump operations. (sentence removed).

#### Intervention

Tranexamic acid (TXA) was the intervention in this study. Both topical and intravenous application of TXA were considered.

#### Comparison

Only randomized controlled trials(RCTs)comparing TXA with placebo were included in this meta-analysis. Head to head trials comparing TXA with other fibrinolytic medications were excluded from analysis.

#### Outcome

Primary outcomes were transfusion rate (the proportion of patients underwent blood transfusion during hospital stay) and transfusion volume (the amounts of blood transfused during hospital stay). Secondary outcomes were post-operative blood loss, re-operation rate, mortality during hospital stay, post-operative complications, including seizure, stroke, myocardial infarction, pulmonary embolism and renal dysfunction.

### Search methods for identification of studies

#### Electronic searches

MEDLINE, EMBASE, and the Cochrane Central Register of Controlled Trials (CENTRAL) were searched systematically for relevant studies. We did not limit the search by language or publication state. Searches in all three databases were searched until 2018/12/31. The methodology was developed from the Preferred Reporting Items for Systematic Reviews and Meta-Analyses (PRISMA) statemen. Details for search strategies were listed in Additional file 1.

#### Searching other resources

We used Google (including Google Scholar) to do comprehensive research on the Internet. We also scanned the reference lists of identified articles and related reviews for relevant studies.

### Data collection and analysis

#### Study selection

The titles and abstracts identified in the electronic search were independently screened by two authors to identify trials that met previously defined inclusion criteria.

#### Data extraction and management

Two authors independently extracted study characteristics and outcomes. Data on the following items were recorded: type of surgery, usage of cardiopulmonary bypass (CPB), presence or absence of a transfusion protocol, method of TXA administration (topical or intravenous, continuous or bolus), drug dosage, number of patients in the experiment and control group, and the outcome measures of interest. The discrepancy was resolved by discussion.

#### Risk of bias assessment

All included studies were assessed for methodological quality by two authors independently. We used the Cochrane Collaboration’s tool for assessing risk of bias (Version 5.1.0) as evaluation criteria. The following domains were assessed for each study: random sequence generation, allocation concealment, blinding of participants and personnel, blinding of outcome assessment, incomplete outcome data addressed and selective reporting. Each domain for every included study was graded into three categories: low risk of bias, unclear risk of bias and high risk of bias.

#### Subgroup analysis and investigation of heterogeneity

Analysis of a-priori subgroups was performed according to the following factors: type of surgery, usage of cardiopulmonary bypass (CPB), intravenous or topical application of TXA, bolus or continuous infusion of TXA, and TXA dosage.

#### Statistical analysis

For dichotomous outcomes (transfusion rate, re-operation rate, incidence of adverse effects), we calculated relative risk (RR) with 95% confidence interval (CI). For continuous outcomes (post-operative blood loss or transfusion volume) reported as mean and standard deviation, the mean difference (MD) for the pooled estimates with 95% CI was calculated. The fixed-effect model was used for analysis with no heterogeneity(I^2^ = 0), and random-effect model was used for analysis with heterogeneity (I^2^>0%). For outcomes with heterogeneity, an effort was made to identify its source, mainly through subgroup analysis. A *P*-value less than or equal to 0.05 for the Q statistic was used to define statistically significant heterogeneity. Statistical heterogeneity was also assessed using the I^2^ test. I^2^ = 0–40%: heterogeneity might not be important; I^2^ = 30–60%: may represent moderate heterogeneity; I^2^ = 50 to 90%: may represent substantial heterogeneity; I^2^ = 75 to 100%: considerable heterogeneity. *P* < 0.05 (2-sided) was considered statistically significant for the hypothesis testing. The publication bias was visualized by symmetry of funnel plot. All statistical analyses were performed in RevMan (version5.0; Cochrane Collaboration, Oxford, UK) and Stata (version 9.0; Stata Corporation, College Station, TX).

## Results

### Description of studies

#### Search results

**Fig. 1:**
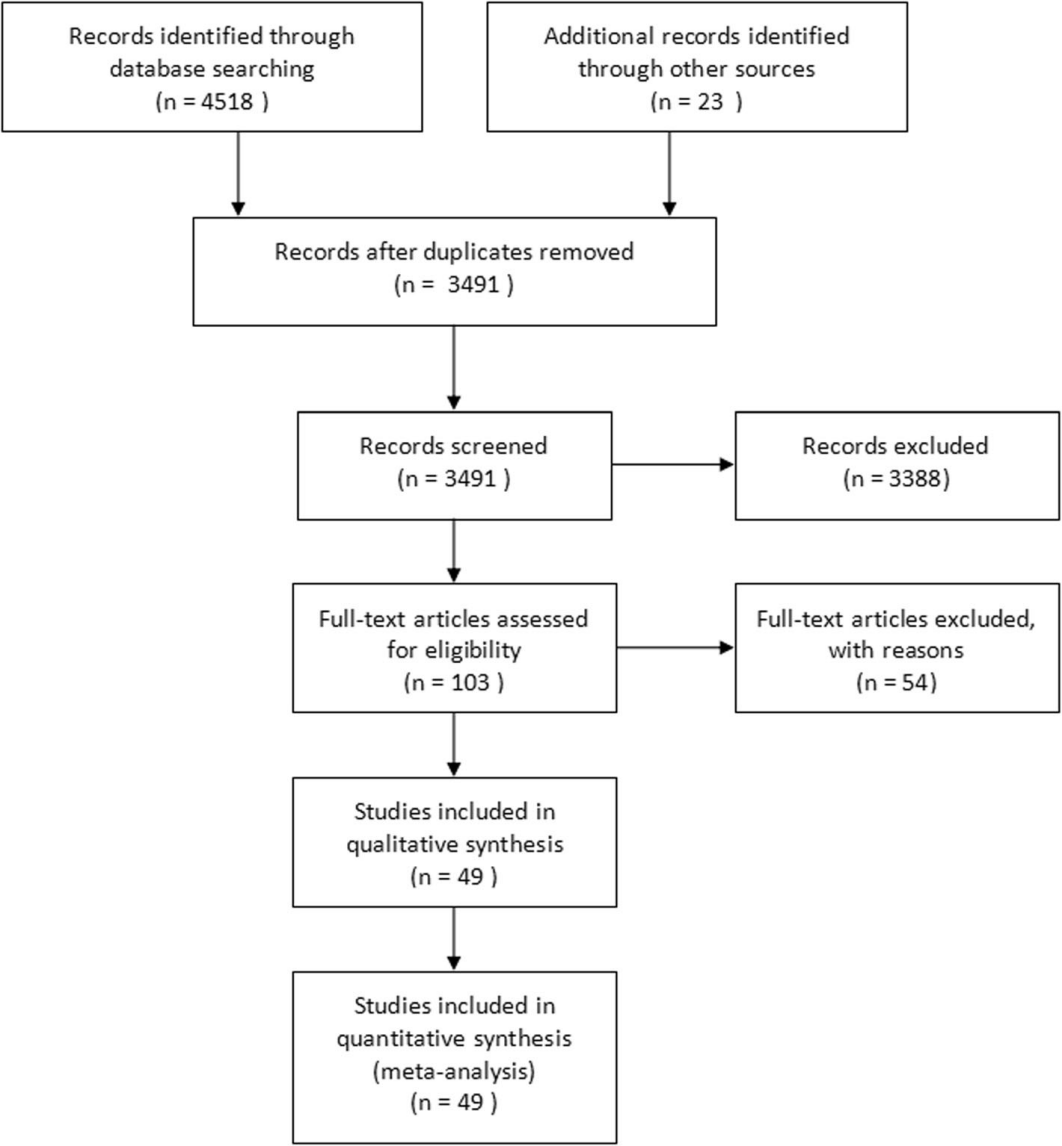
PRISMA diagram showing article selection for the review

We identified 4541 results according to the search strategy described above. The detailed search process was provided in Additional file 1. After assessing 103 full-text articles for eligibility, 49 of them were finally included in the meta-analysis [6, 12–59] (Fig. 1).Major exclusions were provided in Additional file 2.

#### Included studies

We included 49 studies with a total of 10,591 participants in this meta-analysis. The detailed characteristics of all included studies were listed in Additional file 3 One thing worth noticing was that intravenous drug administration varied significantly between trials on dosage regimen. There were mainly two types of intravenous administration methods. One was bolus infusion alone (14 trials) and the other was bolus injection followed by continuous infusion (22 trials). Since these two delivery methods varied significantly in pharmacokinetics, we decided the cutoff value for high and low dose TXA separately. According to what’s mentioned in the background, we defined<50 mg/kg of TXA as low dose for bolus injection, and ≤ 10 mg/kg + 1 mg/kg/h TXA as low dose for bolus plus continuous infusion.

#### Risk of bias in included studies

**Fig. 2:**
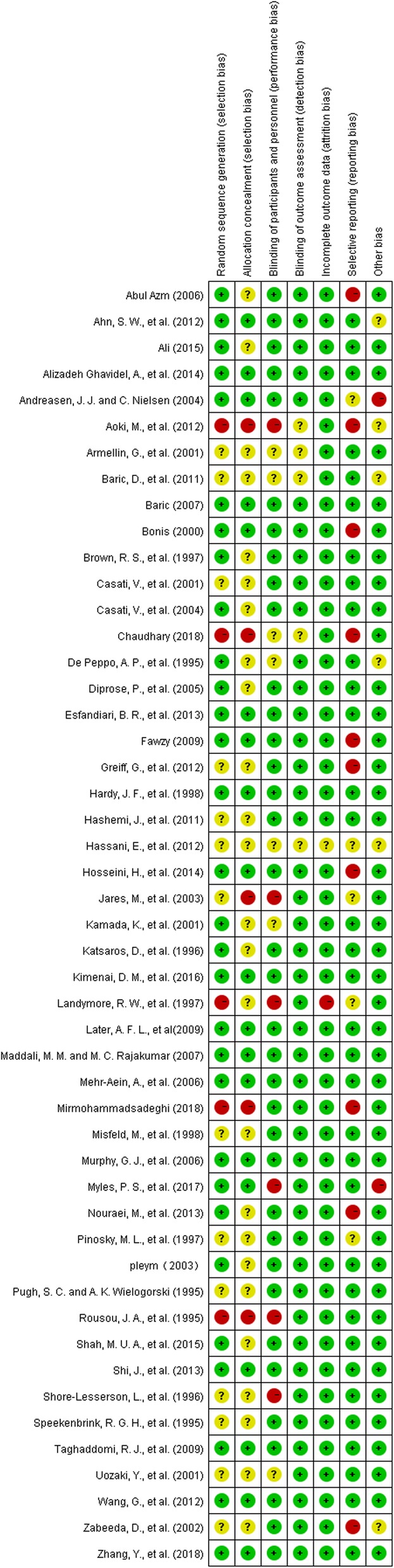
risk of bias graph

**Fig. 3:**
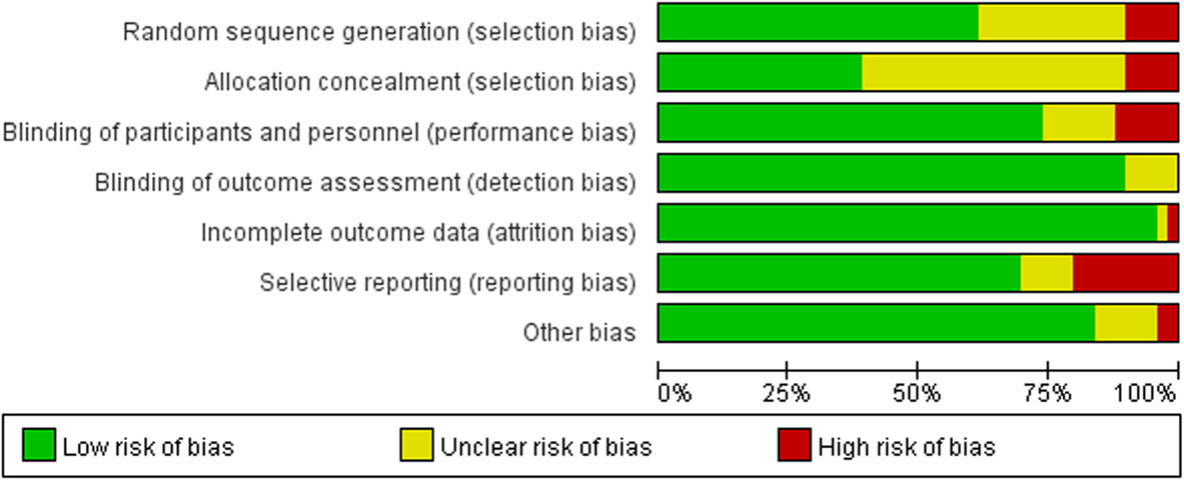
Risk of bias summary

Details regarding the performance of the studies against each domain were presented in the Risk of bias graph (Fig. 2). Additionally, a visual summary of judgement s about each methodological quality item for each included trial was shown in Fig. 3.

Most of the trials included had low risk of bias on random sequence generation, blinding, incomplete outcome data and selective reporting. Selective reporting turned out to be the main risk of bias in this meta-analysis. We rated 33 trials as having low risk of selective reporting, 11 trials as having high risk of selective reporting and 5 trial as having unclear risk of selective reporting. Typical bias for selective reporting was that some studies failed to report transfusion rate while only reporting transfusion volume or vice versa. There was a risk that insignificant data were concealed, and results were thus misrepresented. Also, many of the trials failed to report information on allocation concealment and were rated to be have unclear risk of bias for this item. In summary, there were 13 trials with high risk of bias, 13 trials with low risk of bias and 23 studies with unclear risk of bias.

## Effects of interventions

### Effects and adverse outcomes

#### Transfusion rate

**Fig. 4:**
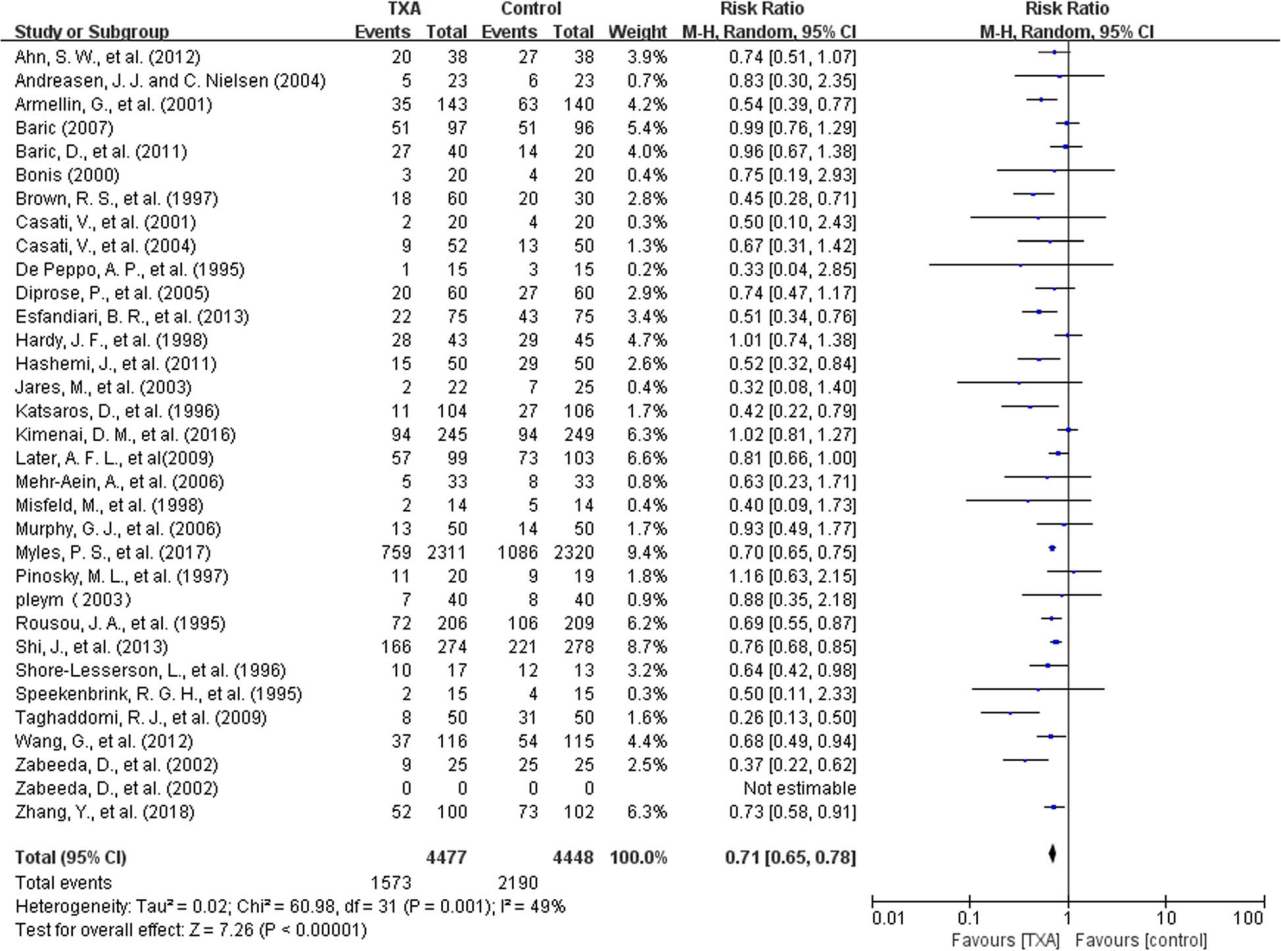
TXA vs Control-transfusion rate

There were 31 trials with 8925 patients that reported data on the number of patients exposed to blood transfusion. The use of TXA significantly reduce the need for allogeneic blood transfusion by a relative 29% (RR 0.71, 95% CI 0.65 to 0.78, P<0.00001). The overall transfusion rate was 35%(1573/4477) for patients using TXA and 49%(2190/4408) for patients in the control group, with a reduction in absolute risk of 0.14 (95%CI 0.12, 0.16). Heterogeneity between these trials was moderate (Chi^2^ = 60.98, df = 31, *P* = 0.001; I^2^ = 49%) (Fig. 4).

#### Transfusion volume

**Fig. 5:**
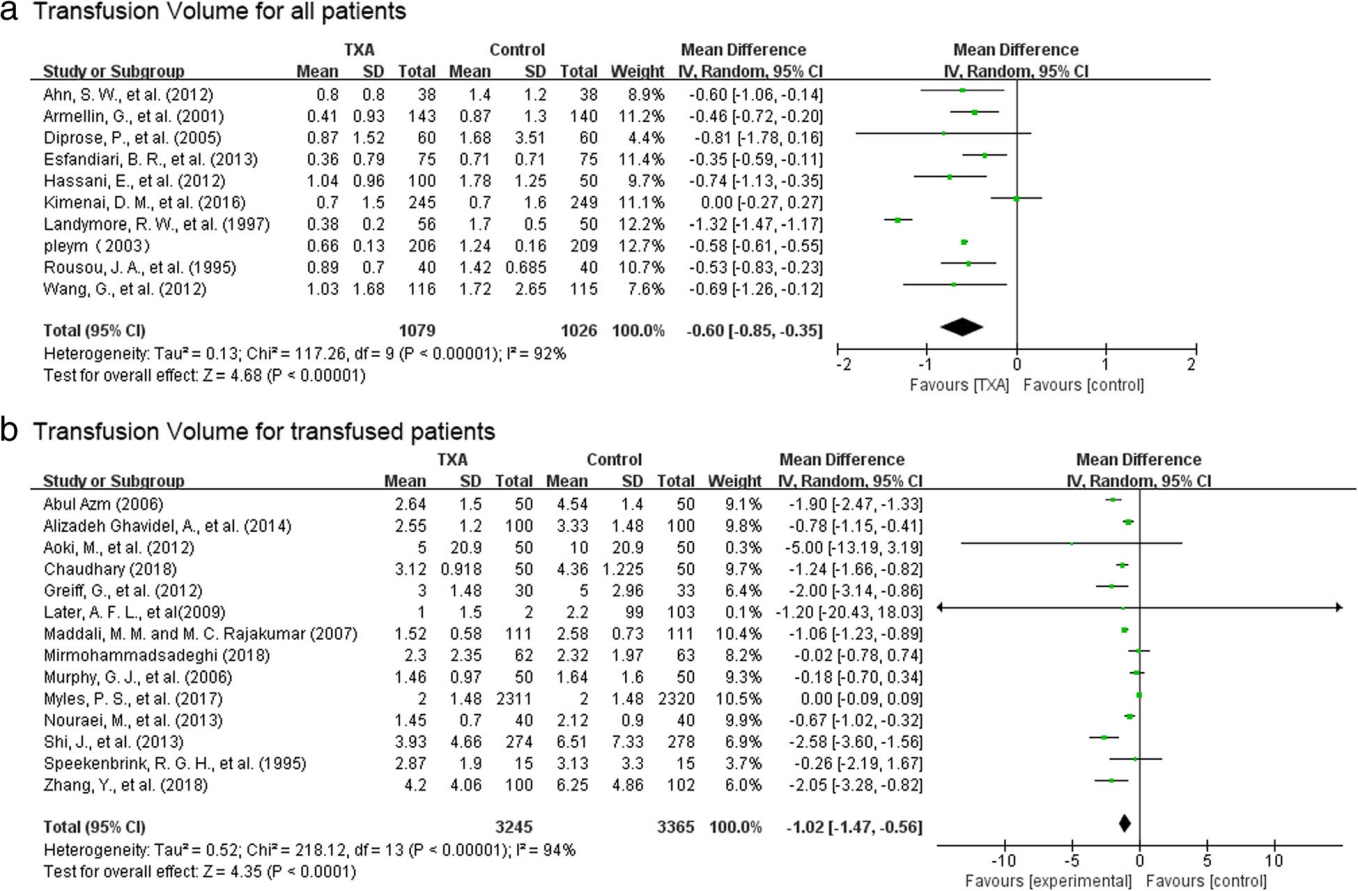
TXA vs control—transfusion volume; **a** transfusion volume for all patients, **b** transfusion volume for transfused patients

There were 10 trials with 2105 patients that reported data on the volume of blood transfused in all patients. The use of TXA resulted in 0.6 units reduction of allogeneic blood per patient (MD-0.60 units, 95% CI − 0.85 to − 0.35 unit, P<0.00001). Heterogeneity between these trials was significant (Chi^2^ = 117.26, df = 9, P<0.00001; I^2^ = 92%) (Fig. 5a).

There were 14 trials with 6610 patients that provided data on the volume of blood transfused in those patients transfused. The use of TXA resulted in a 1.02 units reduction of blood transfusion per patient (MD − 1.02 units, 95% CI − 1.47 to − 0.56 units, P<0.00001). Heterogeneity between trials was significant (Chi^2^ = 218.12, df = 13, P<0.00001; I^2^ = 94%) (Fig. 5b).

#### Post-operative blood loss

**Fig. 6:**
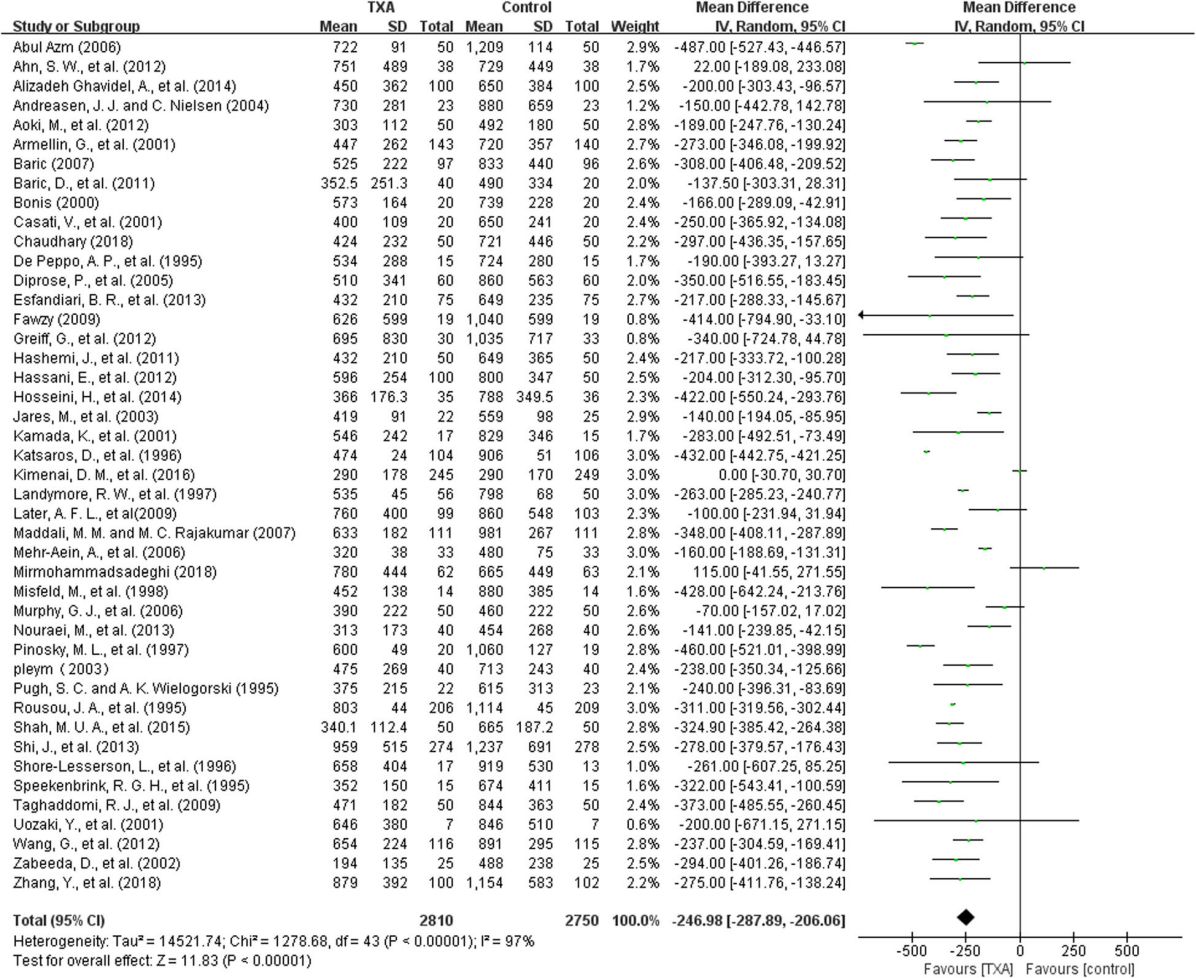
TXA vs control-post-operative blood loss

There were 44 trials with 5560 patients that reported post-operative blood loss. On average, TXA treatment reduced post-operative blood loss by around 247 ml per patient compared to control (MD − 246.98 ml, 95% CI − 287.89 to − 206.06 ml, P<0.00001). Heterogeneity between these trials was significant (Chi^2^ = 1278.68, df = 43, *P* < 0.00001; I^2^ = 97%) (Fig. 6).

##### Re-operation

**Fig. 7:**
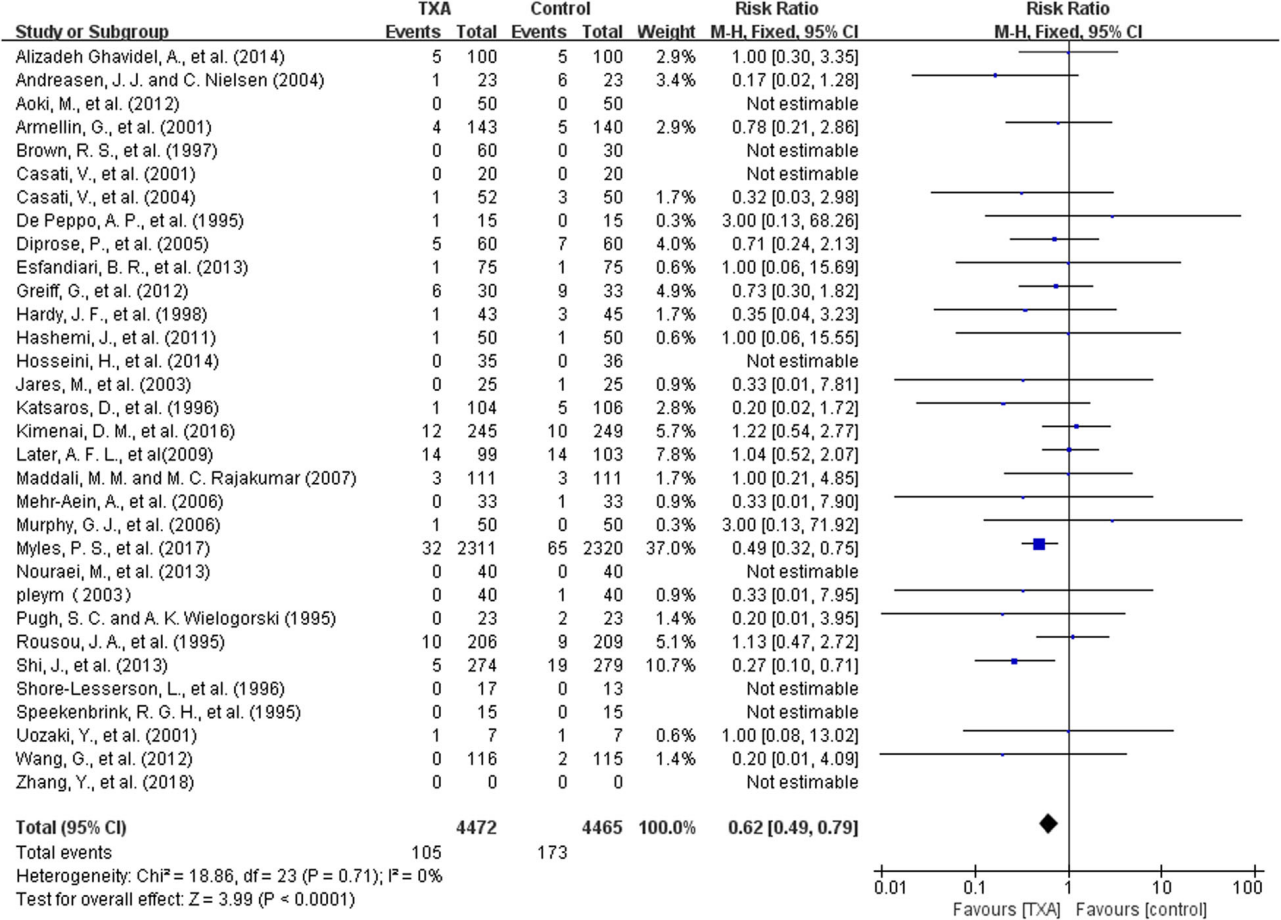
TXA vs control-reoperation rate

There were 32 trials with 8937 patients that reported data on reoperation for all reasons. The use of TXA significantly decrease the risk of reoperation by 38% (RR 0.62, 95% CI 0.49 to 0.79, P<0.0001). The overall reoperation rate was 2.4% (105/4472) for patients using TXA and 3.9% (173/4465) for patients in the control group, with a reduction in absolute risk of 0.01 (95%CI 0.01, 0.02). Heterogeneity between these trials were low (Chi^2^ = 18.86, df = 23, *P* = 0.71; I^2^ = 0%) (Fig. 7).

#### Seizure

**Fig. 8:**
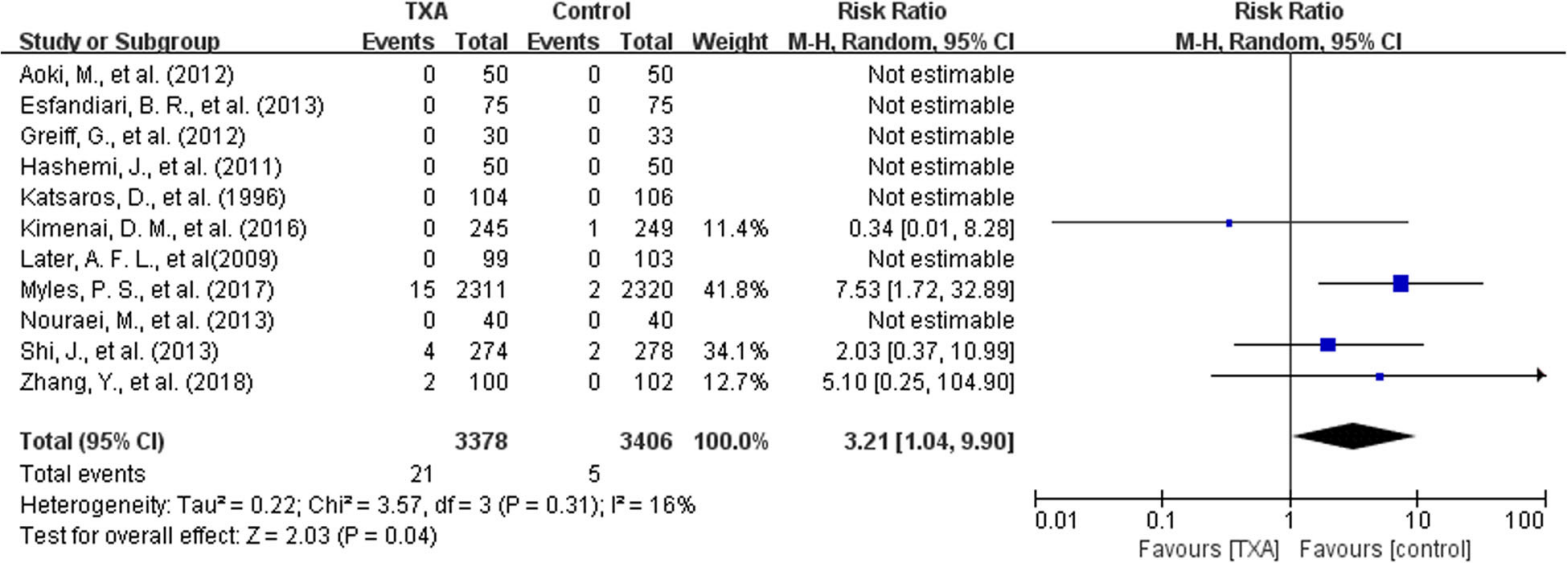
TXA vs control-seizure

There were 11 trials with 6784 patients that reported data for seizure. The use of TXA was associated with a 3.21 folds increase in the risk of seizure (RR 3.21, 95% CI 1.04 to 9.90, *P* = 0.04). The overall rate of seizure attack was 0.62%(21/3378) for patients using TXA and 0.15%(5/3406) for patients in the control group, with an increase in absolute risk of 0.00 (95%CI 0.00, 0.01).Mild heterogeneity existed between these trials (Chi^2^ = 3.57, df = 3, *P* = 0.31; I^2^ = 16%) (Fig. 8).

#### Mortality, thrombotic events and renal injury

As is shown in Table 1, there was no significant difference between TXA and control group in mortality (29 studies, 8970 patients, *P* = 0.20), stroke (32 studies, 9257 patients, *P* = 0.50), myocardial infarction (32 studies, 8688 patients, *P* = 0.67), pulmonary embolism (18 studies, 6587 patients, *P* = 0.60) or renal dysfunction (19 studies, 7210 patients, *P* = 0.92). All of these results were of low heterogeneity with I2 = 0%. All the detailed information and forest plot of these results were proved in Additional file 4: Figure S1, Additional file 5: Figure S2, Additional file 6: Figure S3, Additional file 7: Figure S4 and Additional file 8: Figure S5.

**Table 1:** TXA vs Control for mortality and thrombotic events

Endpoints	Number of studies/reference	Overall participants	Effect estimate (RR, 95% CI)	P	Heterogeneity(I^2^)
Mortality	29	8907	0.78(0.54, 1.14)	0.20	0%
Stroke	32	9257	0.88(0.61, 1.28)	0.50	0%
Myocardial infarction	32	8688	0.89(0.77, 1.04)	0.67	0%
Pulmonary embolism	18	6587	1.08(0.59,2.00)	0.60	0%
Renal dysfunction	19	7210	0.99(0.77, 1.27)	0.92	0%

### Subgroup analysis

For primary outcomes, subgroup analysis was done only on transfusion rate, given the fact that too few studies reported results on transfusion volume (either for all patients or for transfused patients) to conduct subgroup evaluation.

For secondary outcomes, subgroup analysis was done merely on seizure which was the only adverse event showing significant difference between TXA and control group.

#### Transfusion rate——on and off-pump surgeries

There were 7 off-pump trials and 23 on-pump trials that reported data for transfusion rate. The use of TXA significantly reduce the need for allogeneic blood transfusion by a relative 40% (RR 0.60, 95% CI 0.44 to 0.83, *P* = 0.002) for off-pump trials and 29% (RR 0.71, 95% CI 0.63 to 0.80, P<0.00001) for on-pump trials. Detailed data were provided in Additional file 9: Figure S6.

#### Transfusion rate——types of surgery

There were 22 trials on CABG and 8 trials on other types of heart surgeries that reported data for transfusion rate. The use of TXA significantly reduce the need for allogeneic blood transfusion by a relative 31% (RR 0.69, 95% CI 0.65 to 0.73,P<0.00001) for CABG trials and 15% (RR 0.85, 95% CI 0.74 to 0.96, *P* = 0.01) for trials including other kinds of surgery. Detailed data were provided in Additional file 10: Figure S7.

#### Transfusion rate——intravenous and topical application

There were 28 trials on intravenous (IV) infusion and 4 trials on topical application of TXA that reported data for transfusion rate. For IV infusion, the use of TXA significantly reduce the need for allogeneic blood transfusion by a relative 30% (RR 0.70, 95% CI 0.66 to 0.74, P<0.00001). Heterogeneity between these trials was moderate (Chi^2^ = 29.28, df = 26, *P* = 0.30; I^2^ = 11%).

For topical application, the use of TXA did not reduce the need for allogeneic blood transfusion (RR 1.02, 95% CI 0.87 to 1.20, *P* = 0.76). Heterogeneity between these trials was low (Chi^2^ = 2.18, df = 3, *P* = 0.54; I^2^ = 0%) (Additional file 11: Figure S8).

#### Transfusion rate——bolus and bolus plus continuous infusion

There were 10 trials on bolus infusion and 18 trials on bolus plus continuous infusion of TXA that reported data for transfusion rate. In the 18 trials with 2310 patients that had bolus plus continuous infusion of TXA, the use of TXA significantly reduce the need for allogeneic blood transfusion by a relative 30% (RR 0.70, 95% CI 0.65 to 0.76, P<0.00001). Heterogeneity between these trials was moderate (Chi^2^ = 18.92, df = 17, *P* = 0.33; I^2^ = 10%).

**Fig. 9:**
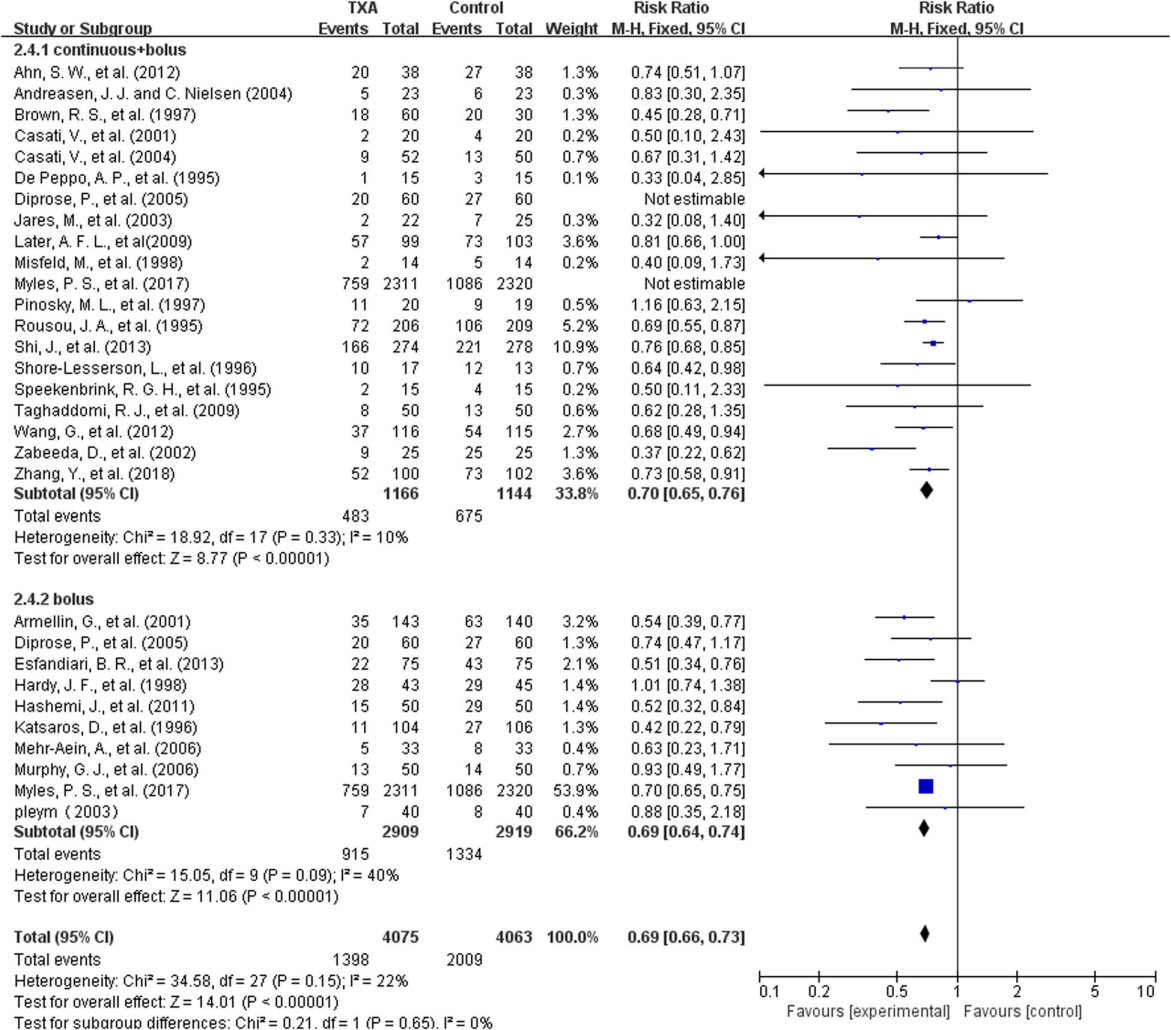
bolus and bolus plus continuous infusion-transfusion rate

In the 10 trials with 5828 patients on bolus injection of TXA, the use of TXA significantly reduce the need for allogeneic blood transfusion by a relative 31% (RR 0.69, 95% CI 0.64 to 0.74, P<0.00001). Heterogeneity between these trials was moderate (Chi^2^ = 15.05, df = 9, *P* = 0.09; I^2^ = 40%) (Fig. 9).

#### Transfusion rate——high and low dose regimen

For bolus plus continuous infusion, there were 6 trials with low-dose regimen (≤10 mg/kg + 1 mg/kg/h) and 12 trials with high-dose regimen. For bolus delivery alone, there were 5 trials with low-dose regimen(<50 mg/kg) and 4 trials with high-dose regimen.

For bolus plus continuous infusion, in the 6 low-dosage trials with 265 patients, the use of TXA significantly reduce the need for allogeneic blood transfusion by a relative 50% (RR 0.50, 95% CI 0.38 to 0.67, P<0.00001). Heterogeneity between these trials was moderate (Chi^2^ = 8.94, df = 5, *P* = 0.11; I^2^ = 44%). In the 12 high-dosage trials with 2043 patients, the use of TXA significantly reduce the need for allogeneic blood transfusion by a relative 29% (RR 0.71, 95% CI 0.65 to 0.71, *P*<0.00001). Heterogeneity between trials was moderate (Chi^2^ = 13.84, df = 11, *P* = 0.24; I^2^ = 21%) .

**Fig. 10:**
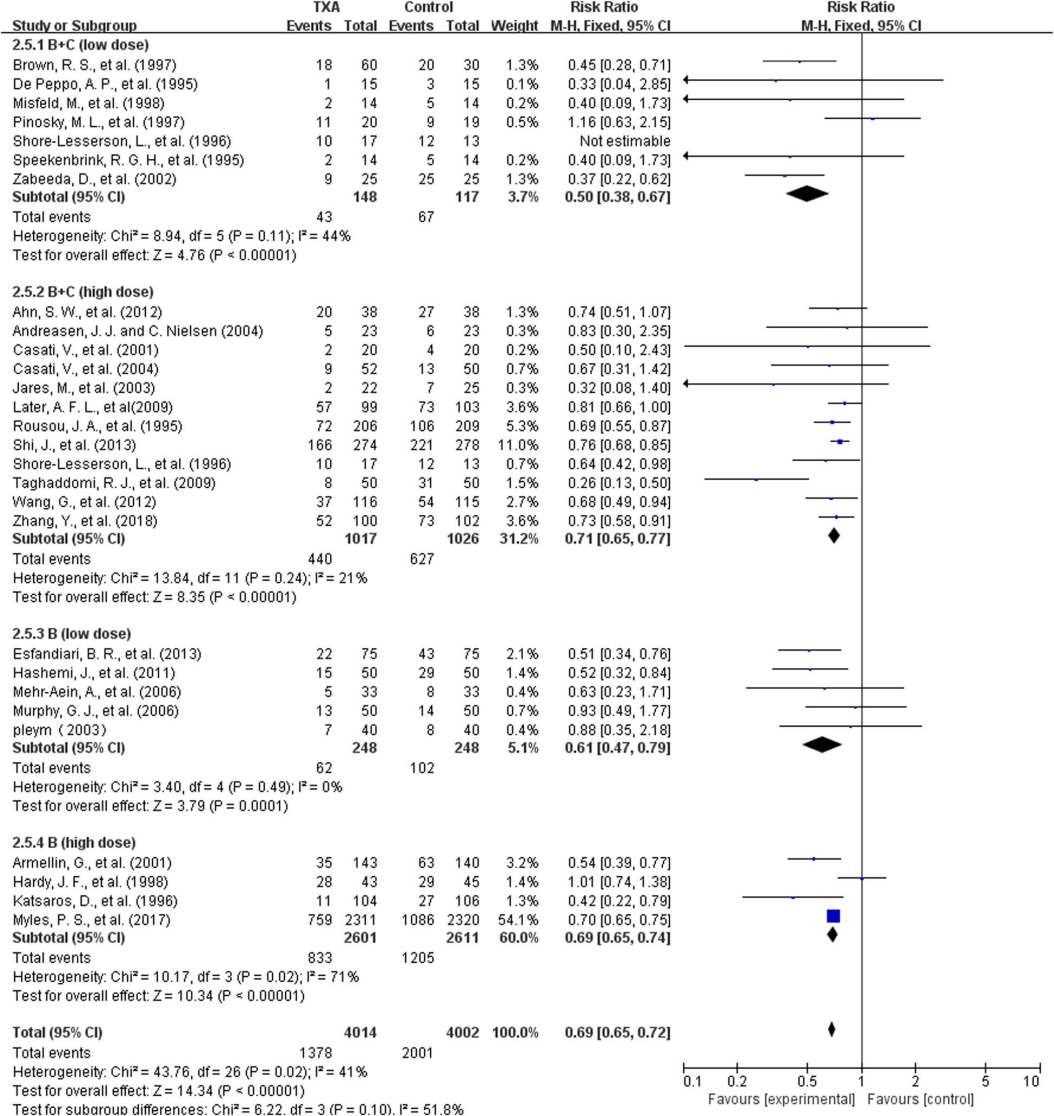
high and low dose regimen-transfusion rate

For bolus infusion alone, in the 5 low-dosage trials with 496 patients, the use of TXA significantly reduced the need for allogeneic blood transfusion by a relative 39% (RR 0.61, 95% CI 0.47 to 0.79, *P* = 0.0001).Heterogeneity between these trials was low(Chi^2^ = 3.40, df = 4, *P* = 0.49; I^2^ = 0%).In the 4 high-dosage trials with 5212 patients, the use of TXA significantly reduced the need for allogeneic blood transfusion by 31%(RR 0.69, 95% CI 0.65 to 0.74, P<0.00001). Heterogeneity between these trials was significant (Chi^2^ = 10.17, df = 3, *P* = 0.02; I^2^ = 71%) (Fig. 10).

#### Seizure-high and low dose regimen

Among the 49 studies included, only 8 trials provided data on seizure attack. Since there were too few studies on seizure, we combined bolus with bolus plus continuous infusion together when analyzing high and low dose regimen(<50 mg/kg or ≤ 10 mg/kg + 1 mg/kg/h). Five trials on high dose regimen and 3 trials on low dose regimen were analyzed.

**Fig. 11:**
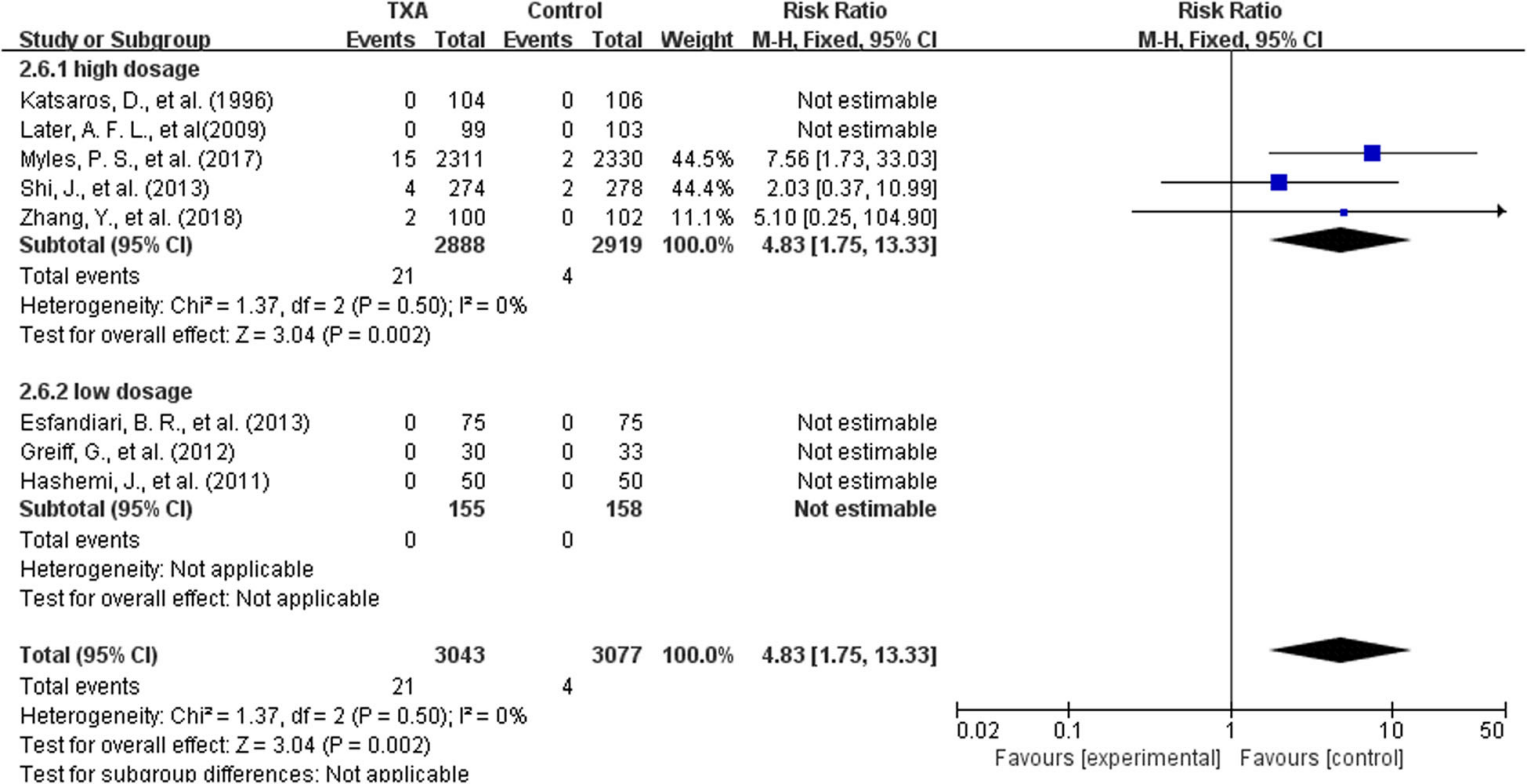
high and low dose regimen-seizure

In the 5 high-dose trials with 5807 patients, the use of high-dosage TXA significantly increased the risk of seizure attack by 4.83 times (RR 4.83, 95% CI 1.75 to 13.33, *P* = 0.002). Heterogeneity between these trials was low (Chi^2^ = 1.37, df = 2, *P* = 0.50; I^2^ = 0%). In the 3 low-dosage trials with 313 patients, no seizure occurred in any of the trials, and the impact of low-dosage TXA on seizure attack could not be assessed (Fig. 11).

### Impact of trial quality

We did a subgroup analysis on transfusion rate for studies with low and unclear risk of bias only and found that the use of TXA reduced the rate of allogeneic blood transfusion by a relative 29% (RR 0.71, 95% CI 0.68 to 0.75, P<0.00001). Heterogeneity between these trials was significant (Chi^2^ = 59.50, df = 27, *P* = 0.0003; I^2^ = 55%) (Additional file 12: Figure S9). This result was largely the same with results including all studies. Excluding trials with high risk of bias didn’t make a difference.

### Publication bias

Funnel plot comparing TXA with control group concerning transfusion rate was provided in Additional file 13: Figure S10. No obvious asymmetry was seen from the funnel plot, and thus no publication bias was detected.

## Discussion

In this meta-analysis, we strengthened the results that TXA significantly reduced blood loss, transfusion requirement and re-operation rate in adult cardiac surgery. In addition to updating previous findings, we tried to answer three important questions: whether TXA is effective for all kinds of elective heart surgeries on adults, whether TXA leads to adverse effects, what’s the preferable dose regimen and delivery method for TXA.

We found TXA to be effective for both off-pump and on-pump operations. Although off-pump patients didn’t undergo CPB, they were inevitability exposed be heparin, protamine and even greater surgical trauma than on-pump patients, and we found that these patients could benefit as much as, if not more than on-pump patient from TXA.

TXA did not show any trend to increase the risk of thrombotic events including myocardial infarction, stroke and pulmonary embolism, and wasn’t associated with renal injury. But TXA significantly increased the risk of seizure, which was a result well worth attention. According to our findings, seizure was a rare event in patients underwent cardiac surgeries. There were 11 studies that reported data on epileptic attack and 7 of them reported no attack in both TXA and control groups. In the other 4 studies, seizure happened at a rate of 0.20–1.08%. Given the rare nature of seizure attack, we consider it valuable to look at results from retrospective observational studies. In an observational study of 12,195 patients underwent cardiac surgery, none of the 886 patients in the control group without TXA administration had seizure and 80 out of 9642 patients (0.83%) with TXA delivery had seizure [60]. Takagai et al. did a meta-analysis on all kinds of researches concerning the association between TXA and seizure in cardiac surgeries [61]. They identified 16 studies with 45,235 patients, and demonstrated that TXA therapy was associated with a statistically significant increase in seizures incidence (OR = 4.13; 95% CI: 2.59 to 6.57; *P* < 0.00001). A subgroup analysis indicated a significant increase seizure in all subgroups of 5 RCTs, 5 adjusted observational studies, and 6 unadjusted observational studies with no statistically significant subgroup difference (*P* = 0.36; I^2^ = 1.5%).

We found that intravenous delivery of TXA significantly reduced transfusion rate by 30%, while topical application did not show any signs to reduce transfusion requirement. This result can be further confirmed by several studies comparing intravenous plus topical use of TXA with intravenous application alone. In a RCT conducted by Taksaudom et al. [62], 1 g of topical TXA was poured into the pericardial cavity in addition to 1 mg/kg/h of intravenous infusion in the experiment group, and only 1 mg/kg/h of intravenous infusion was applied in the control group. No significant reduction was seen in post-operative blood loss, and transfusion rate was even higher in the experiment group (75.6% vs 70.6%). The same happened for another RCT conducted by Spegar et al. in which 2.5 g topical TXA was applied in addition to 1 g + 400 mg/h of intravenous infusion [63]. According to existing evidence, topical TXA was not effective enough to reduce bleeding and transfusion requirements no matter used alone or combined with intravenous application, and further research is needed to justify its use in cardiac procedures.

Both continuous plus bolus and bolus delivery alone significantly reduced transfusion rate compared to the control group, and bolus plus continuous delivery (RR 0.70) did not seem to be more effective than bolus injection (RR 0.69). In a RCT conducted by Imtiaz, et al., 137 CABG patients were randomly assigned to receive either bolus injection or continuous infusion of 30 mg/kg TXA, and no difference were found in blood loss or transfusion requirements [64]. This result was in accordance with our finding that bolus and bolus plus continuous delivery were equally effective. We infer that both delivery methods are able to keep the effect compartment concentration above minimum effective concentration during surgery as long as dosage is enough, but peak concentration may be higher if bolus injection alone is applied which may cause more adverse events. However, this hypothesis needs to be tested by pharmacokinetics studies.

From subgroup analysis, we can conclude that low-dose intravenous TXA, no matter delivered as bolus injection or bolus plus continuous infusion, was effective in reducing transfusion requirements. When comparing with high-dose regimen, lose-dose TXA was at least equally effective, and may be even more effective in reducing transfusion rate. The risk ratio for transfusion rate were 0.50 and 0.61 for low-dose bolus and bolus plus continuous infusion separately, while for high-dose regimen risk ratio was 0.71 and 0.69 separately. As we mentioned above, the use of TXA was significantly associated with an increase in seizure attack. In subgroup analysis, TXA increased the risk of seizure only in patients using high-dose regimen, and none of the patients using low-dose TXA had seizure attack. These evidences give us the basic consumption that low-dose TXA is enough in reducing transfusion requirement and is less likely to cause seizure.

There were several studies that directly compared the effectiveness of high and low-dose regimen of TXA, but the definition of high and low-dose regimen varied significantly among these studies, we thus consider it improper to combine the results together in meta-analysis. Here we did a qualitative other than quantitative analysis of these studies and see if they were in accordance with our basic consumption.

We identified 6 RCTs that compare transfusion rate between high and low dose regimen [65–72], and none of them showed significant decrease in transfusion rate in the high dose group. Fewer studies reported data on transfusion volume, again no significant decrease were found in allogeneic RBC transfusion in the high dose group [66, 70]. These results agreed with our previous findings. In terms of postoperative blood loss, the results were less unanimous. We found 8 RCTs comparing blood loss between high and low dose regimen [65–72], among which 5 studies reported no difference in blood loss [65, 66, 69, 71, 72]. Karski et al. [68] randomly assigned patients to receive 50, 100 or 150 mg/kg of TXA infusion and found that blood loss was significantly higher in the 50 mg/kg group. Jiménez et al. [67] reported blood loss to be significantly reduced in patients who received 80 mg/kg TXA compared with patients who received 40 mg/kg TXA. Sigaut et al. (71)found that blood loss was significantly reduced when 30 mg/kg + 16 mg/kg/h of TXA was infused compared with 10 mg/kg + 1 mg/kg/h of TXA. We identified 3 studies that reported data on seizure [66, 67, 70], and none of them found significant difference between the high and low-dose group, but there was a trend towards more seizure attack. Jiménez et al. [67] reported seizure rate to be 2/80 in the high-dose group and 0/80 in the low dose group. Sigaut et al. [70] found that seizure happened in 3 out of 285 patients in the high dose group and 1 out of 284 patients in the low dose group. In the study conducted by Du et al. [66], seizure rate were 1/87 and 1/88 in high and low dose group separately, and the dose regimen used in their study were 30 mg/kg + 16 mg/kg/h for high-dose and 10 mg/kg + 2 mg/kg/h for low-dose regimen. From these trials which directly compared high and low dose TXA, we can conclude that high dose TXA has little effect in reducing transfusion requirement and tends to cause more seizure attacks, but it may be more effective in reducing blood loss compared to low dose regimen.

Basic researches may explain this different dosage requirements. As we’ve mentioned in the background, TA concentrations required to suppress fibrinolysis and plasmin-induced platelet activation are 10 and 16 μg/ml, respectively [7, 8]. TXA concentration of 10 μg/ml will result in 80% inhibition of tissue plasminogen activator, but in order to achieve 100% inhibition of tissue plasminogen activator, a tranexamic acid concentration of 100 μg/ml is necessary [73]. Another potential mechanism of TXA action is the increase in thrombin formation, which requires concentrations more than 126 μg/ml to be therapeutic [10, 11]. It is likely that low-dose TXA as 10 mg/kg bolus injection followed by 1 mg/kg/h continuous infusion is able to achieve a large proportion of TXA efficacy, and the effect added by high dose regimen isn’t large enough to further decrease transfusion requirements but may reduce blood loss to certain extent.

We noticed that the stratification for risk of bleeding/transfusion may be useful in guiding the dosage of TXA. In the RCT conducted by Sigaut et al. [70], no difference in transfusion requirement was found between high and low dose TXA group, but when subgroup analysis was conducted for patients with high risk of transfusion, a significant decrease in transfusion volume were seen in the high-dose group. In this study, the definition of high-risk of transfusion was as follow: “Patients were considered at high risk for transfusion if they were receiving a dual anti-platelet at any time within 5 days of surgery, or in the following cases: repeat coronary artery bypass graft, repeat valve surgery (replacement or repair), combined coronary artery bypass graft and valve surgery, multiple valve surgery, surgery of the aorta, intracardiac tumor ablation, and surgery for endocarditis. All other cardiac surgery procedures were considered low risk.” We believe that further researches are needed for patients with high-risk of bleeding to decide whether the dosage of TXA should be raised.

Heterogeneity in transfusion rate existed between trials (I^2^ = 49%). From subgroup analysis, we can see that heterogeneity mainly came from intravenous and topical delivery method (intravenous I^2^ = 11%, topical I^2^ = 0%, subgroup differences P<0.00001).

There was significant heterogeneity in transfusion volume (transfusion volume for all patients I^2^ = 92%, transfusion volume for transfused patients I^2^ = 94%) and postoperative blood loss(I^2^ = 97%). Topical and intravenous delivery explained part of the problem, but we believe that heterogeneity mainly came from two reasons. For transfusion volume, different studies varied in measurement of blood, some used ml and some used blood unit which was different depending on different countries. For blood loss, studies varied in the time point in measuring postoperative blood loss, form 4 h to the several days. These sources of heterogeneity were hard to solve by subgroup analysis, and we thus used the random-effect model to estimate the average effect of TXA delivery on these outcomes.

No heterogeneity existed concerning adverse events including reoperation rate, mortality, stroke, myocardial infarction, pulmonary embolism and renal dysfunction. Mild heterogeneity existed concerning seizure(I^2^ = 16%), and we’ve identified from subgroup analysis that this heterogeneity came from different dose regimen (high-dose group I^2^ = 0%).

Sensitivity analysis showed that excluding trials with high risk of bias didn’t make a difference on the main result of transfusion rate. While one thing worth noticing was that the RCT conducted by Myles et al. [6] which had the largest sample size among included studies (sample size 4631) was with unclear risk of bias. We consider this article to have unclear of bias mainly for two reasons. One is that after 1392 patients had been enrolled, they reduced the TXA dosage from 100 mg/kg to 50 mg/kg, and the dose reduction underpowered this trial. The other reason is that the first 2127 participants had not been taking aspirin regularly before the trial or had stopped taking aspirin at least 4 days before surgery, while participants who were subsequently enrolled may or may not have been previously exposed to aspirin therapy. Given its large sample size, we did analysis on rest of the studies with low and unclear risk of bias except Myles study, and found that the use of TXA reduced transfusion rate by a relative 27% (RR 0.73, 95% CI 0.68 to 0.78, P<0.00001). Excluding the trial from Myles et al. didn’t make a difference.

This meta-analysis has several limitations. Some studies only reported transfusion volume without providing data on transfusion rate. We contacted the corresponding authors for missing data, but not much reply was received. Another drawback was that we did not perform a network analysis to compare the effect of high and low-dose regimen, which underpowered our result.

## Conclusions

This meta-analysis provides further evidence that TXA significantly reduces peri-operative blood loss and transfusion requirements in adult cardiac surgery, without increasing the risk of serious adverse events except for seizure. The risk of seizure is only seen in high-dose trials delivered intravenously. High-dose TXA does not further decrease transfusion rate and has a strong tendency to cause more seizure attacks compared to the low-dose TXA. We thus consider low-dose TXA(bolus injection<50 mg/kg, or 10 mg/kg + 1 mg/kg/h)to be more preferable.

## Additional files

Additional file 1: Search strategy. (DOCX 15 kb)

Additional file 2: Major exclusions. (XLSX 12 kb)

Additional file 3: Study characteristics. (XLSX 16 kb)

Additional file 4: **Figure S1.** On and off pump-transfusion rate. (PNG 29 kb)

Additional file 5: **Figure S2.** Different surgery types-transfusion rate. (PNG 29 kb)

Additional file 6: **Figure S3.** IV or topical-transfusion rate. (PNG 29 kb)

Additional file 7: **Figure S4.** Transfusion rate-exclude high risk. (PNG 22 kb)

Additional file 8: **Figure S5.** Mortality. (PNG 20 kb)

Additional file 9: **Figure S6.** PE. (PNG 15 kb)

Additional file 10: **Figure S7.** Strole. (PNG 21 kb)

Additional file 11: **Figure S8.** MI. (PNG 21 kb)

Additional file 12: **Figure S9.** Renal dysfunction. (PNG 16 kb)

Additional file 13: **Figure S10.** Funnel Plot for transfusion rate. (PNG 6 kb)

## Data Availability

The data sets used and/or analyzed during the current study are available from the corresponding author on reasonable request.

## Abbreviations

CPB: Cardiopulmonary bypass
RCTs: Randomized controlled trials
TXA: Tranexamic acid
