# Supplementary material for: Different dose regimes and administration methods of tranexamic acid in cardiac surgery: a meta-analysis of randomized trials
Source: BMC Anesthesiol. 2019 Jul 15;19:129. doi: 10.1186/s12871-019-0772-0 (PMC6631782; doi:10.1186/s12871-019-0772-0)
Supplement: Supplementary file 1 — Search strategy. (DOCX 15 kb) [file 12871_2019_772_MOESM1_ESM.docx]

Search for studies before 2018-12-30

| Medline（Ovid） | | |
| --- | --- | --- |
| 1 | (Tranexamic Acid. mp.) or (exp Tranexamic Acid/) | 10109 |
| 2 | exp Surgery/ | 37424 |
| 3 | (randomized controlled trial.pt.) or (controlled clinical trial.pt.) or (randomized.ab.) or (placebo.ab.) or (randomly.ab.) or (trial.ab.) or (groups.ab.) not (animals.sh. not (humans.sh. and animals.sh.)) | 10752 |
| 4 | 1 and 2 and 3  ((Tranexamic Acid. mp.) or (exp Tranexamic Acid/)) and (exp Surgery/) and ((randomized controlled trial.pt.) or (controlled clinical trial.pt.) or (randomized.ab.) or (placebo.ab.) or (randomly.ab.) or (trial.ab.) or (groups.ab.) not (animals.sh. not (humans.sh. and animals.sh.))) | 1807 |

| CENTRAL | | |
| --- | --- | --- |
| 1 | MeSH descriptor: [Tranexamic Acid] explode all trees | 747 |
| 2 | tranexamic acid (Word variations have been searched) | 1800 |
| 3 | 1 or 2 | 1800 |
| 4 | Surgery (Word variations have been searched) | 179464 |
| 5 | 3 and 4 | 1066 |
| 6 | Limit 5 to “trials” | 994 |

| EMBASE | | |
| --- | --- | --- |
| 1 | 'tranexamic acid'/exp OR 'tranexamic acid' | 11436 |
| 2 | 'surgery'/exp OR 'surgery' | 6149311 |
| 3 | 'clinical trial'/de OR 'controlled clinical trial'/de OR 'controlled study'/de | 340618 |
| 4 | 1 and 2 and 3 | 1357 |

4156（总数）→3491（去重后）→1707（限制为2011年之前的部分）→阅读标题和摘要后初步筛选出69篇可能相关的
