# Supplementary figures and images for: Different dose regimes and administration methods of tranexamic acid in cardiac surgery: a meta-analysis of randomized trials
Source: BMC Anesthesiol. 2019 Jul 15;19:129. doi: 10.1186/s12871-019-0772-0 (PMC6631782; doi:10.1186/s12871-019-0772-0)

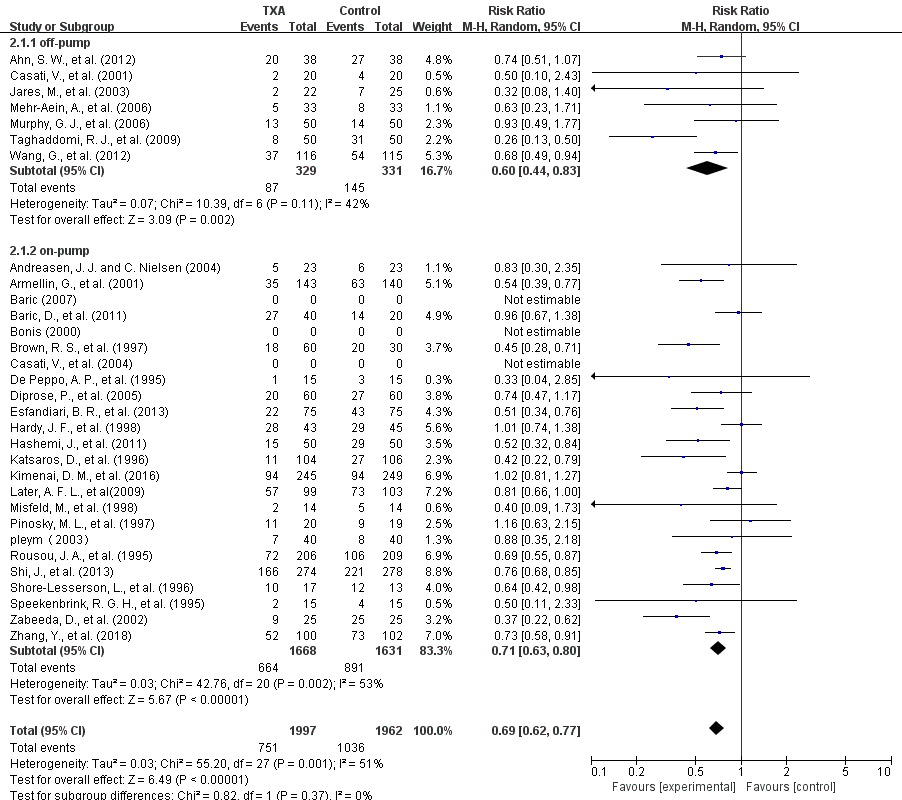

Supplement: Supplementary file 4 — Figure S1. On and off pump-transfusion rate. (PNG 29 kb) [file 12871_2019_772_MOESM4_ESM.png]

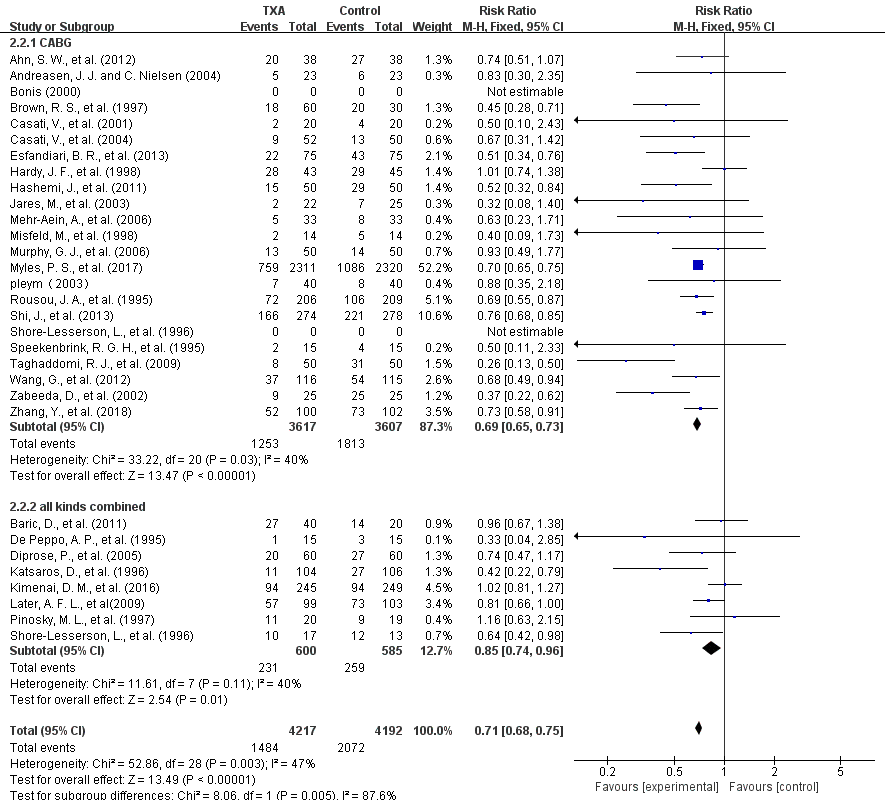

Supplement: Supplementary file 5 — Figure S2. Different surgery types-transfusion rate. (PNG 29 kb) [file 12871_2019_772_MOESM5_ESM.png]

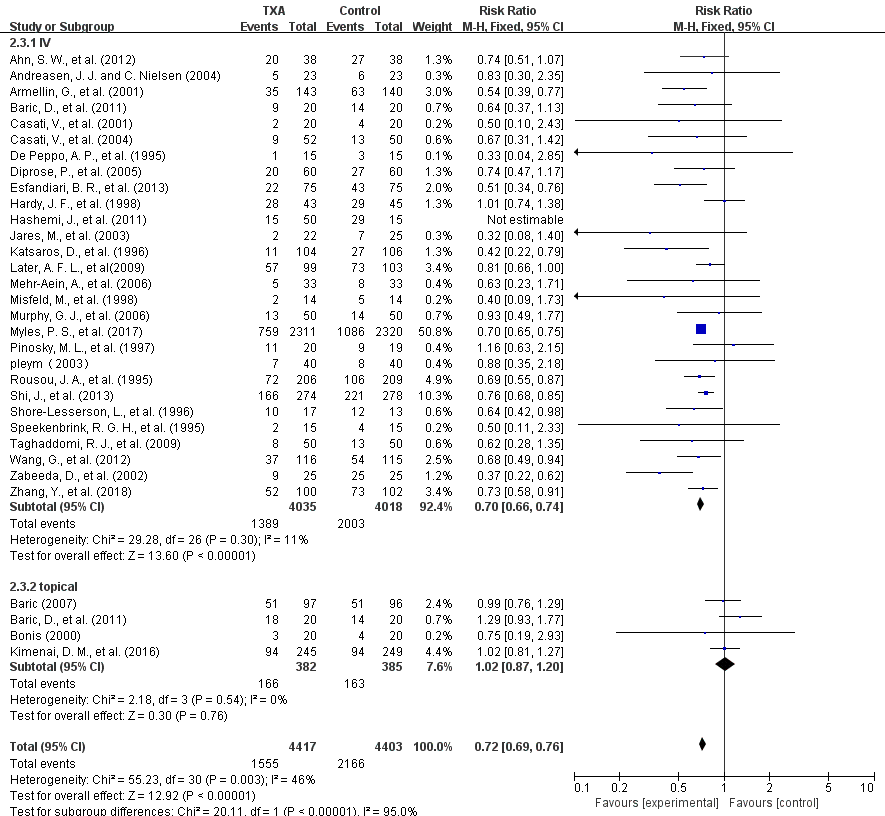

Supplement: Supplementary file 6 — Figure S3. IV or topical-transfusion rate. (PNG 29 kb) [file 12871_2019_772_MOESM6_ESM.png]

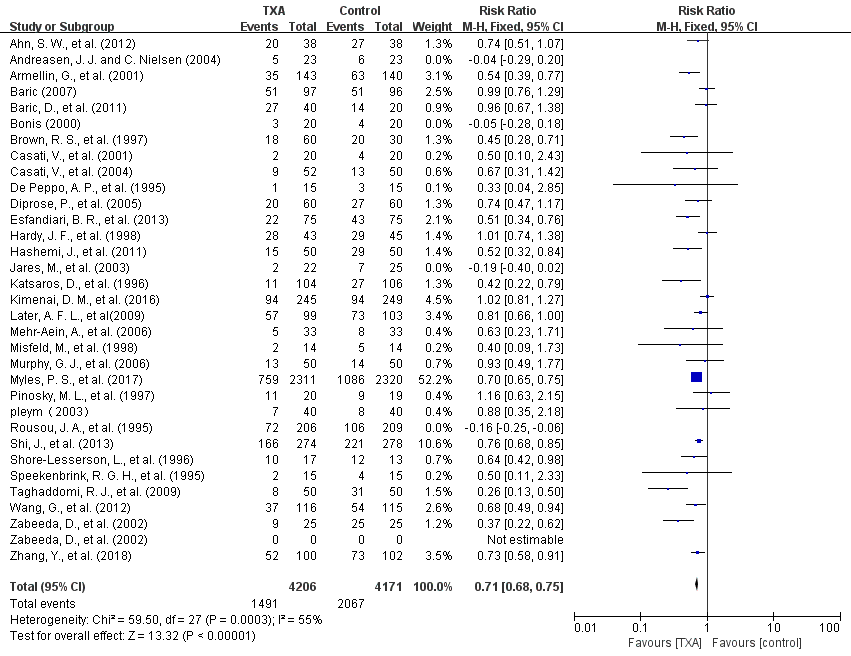

Supplement: Supplementary file 7 — Figure S4. Transfusion rate-exclude high risk. (PNG 22 kb) [file 12871_2019_772_MOESM7_ESM.png]

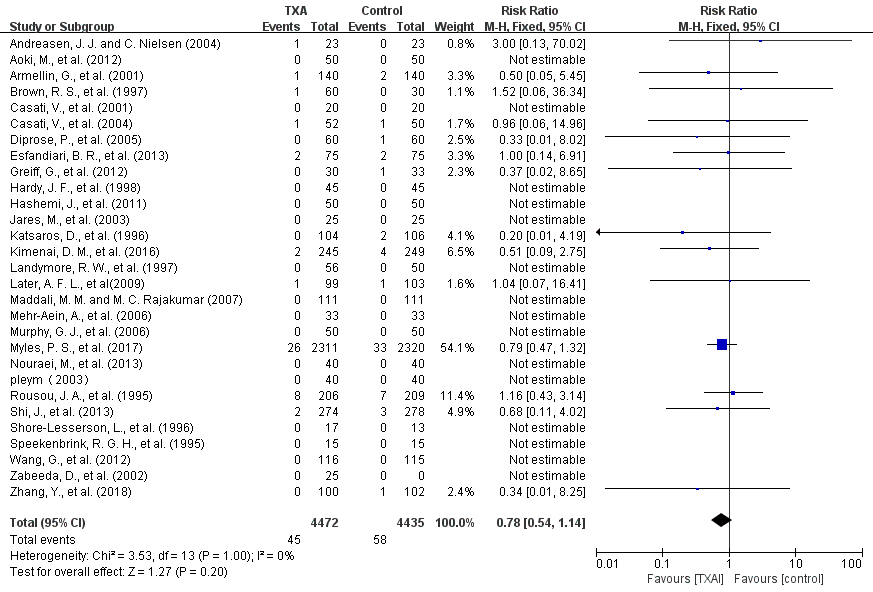

Supplement: Supplementary file 8 — Figure S5. Mortality. (PNG 20 kb) [file 12871_2019_772_MOESM8_ESM.png]

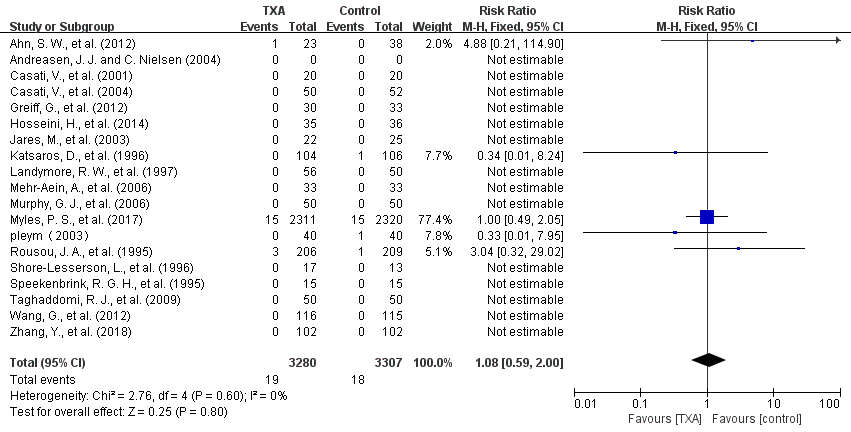

Supplement: Supplementary file 9 — Figure S6. PE. (PNG 15 kb) [file 12871_2019_772_MOESM9_ESM.png]

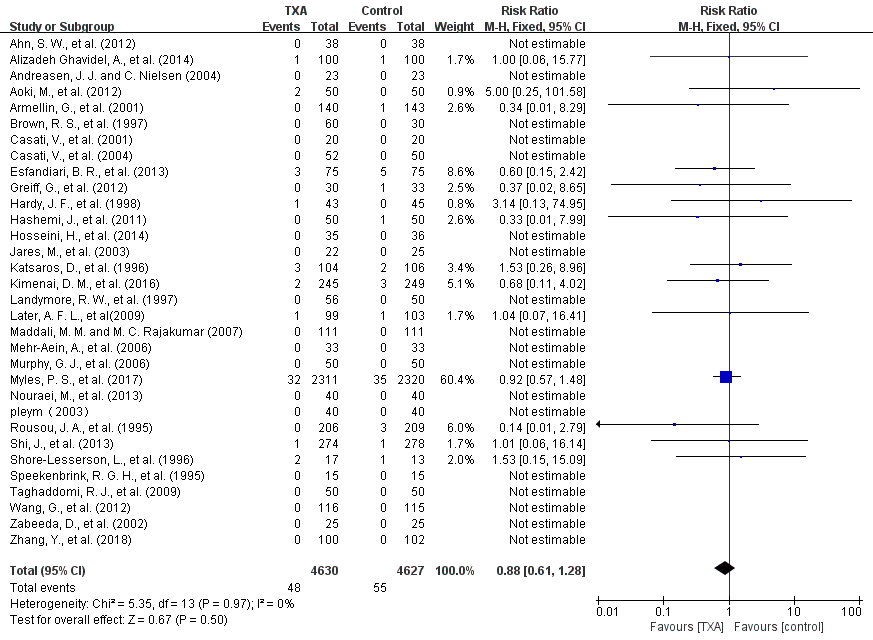

Supplement: Supplementary file 10 — Figure S7. Strole. (PNG 21 kb) [file 12871_2019_772_MOESM10_ESM.png]

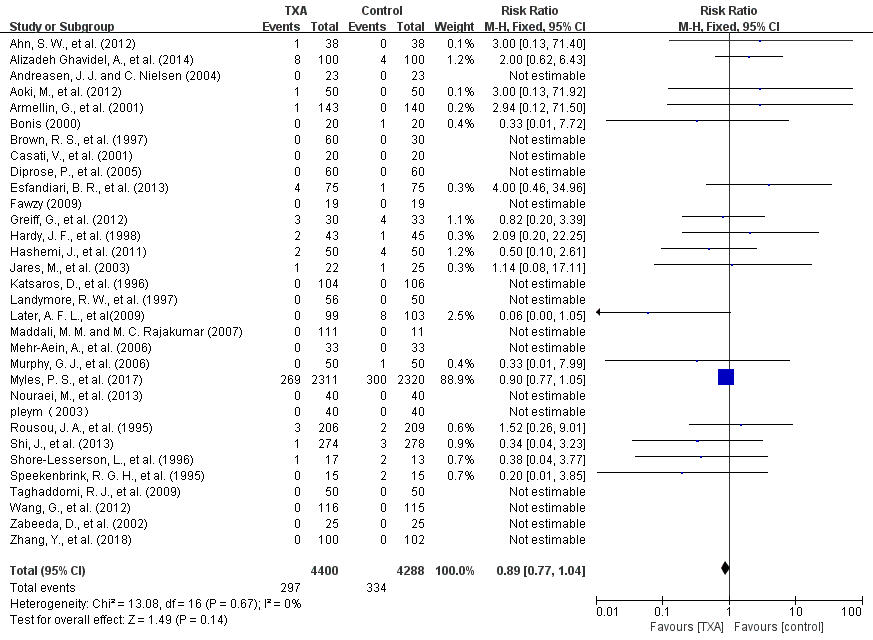

Supplement: Supplementary file 11 — Figure S8. MI. (PNG 21 kb) [file 12871_2019_772_MOESM11_ESM.png]

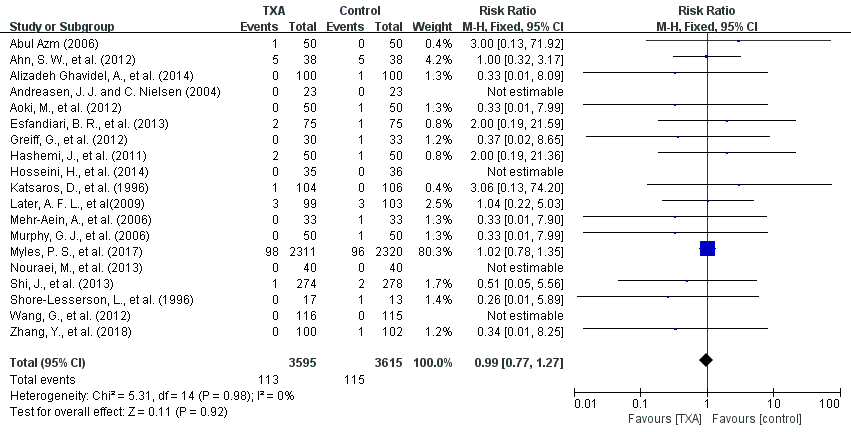

Supplement: Supplementary file 12 — Figure S9. Renal dysfunction. (PNG 16 kb) [file 12871_2019_772_MOESM12_ESM.png]

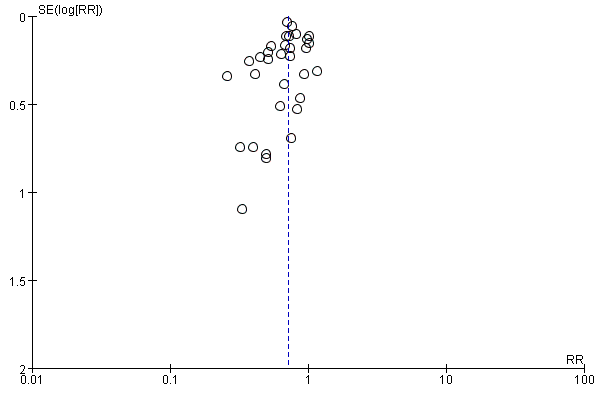

Supplement: Supplementary file 13 — Figure S10. Funnel Plot for transfusion rate. (PNG 6 kb) [file 12871_2019_772_MOESM13_ESM.png]
